# EDUCATION, OCCUPATIONAL ENVIRONMENT, AND COGNITIVE DECLINE IN LATE ADULTHOOD

**DOI:** 10.1093/geroni/igae098.0277

**Published:** 2024-12-31

**Authors:** Katy (Qiuchang) Cao, Dawn Carr, Miles Taylor

**Affiliations:** Florida State University, Tallahassee, Florida, United States; Florida State University, Tallahassee, Florida, United States; Florida State University, Tallahassee, Florida, United States

## Abstract

Education and occupational environment shape cognitive decline and Alzheimer’s Disease and Related dementias (ADRDs) in late adulthood. However, few studies have explored both factors simultaneously. This study aims to (1) Identify occupational environmental factors impacting cognitive decline and ADRD; (2) Analyze the separate or combined contributions of education and occupational environment to cognitive decline and ADRD; (3) Identify whether occupational environment mediates the association between education and cognitive decline/ADRD in late life. Structural Equation Modeling was conducted in Mplus using nationally representative data from the Health and Retirement Study (HRS) and Occupation Information Network (O*NET) linked dataset, incorporating new race/ethnicity adjusted ADRD measures. Confirmatory Factor Analyses suggested that hazardous exposure and cognitive complexity capture two separate but connected components of the occupational environment. Both the measurement (N=7301) and structural model (N=6345) demonstrated good model fit (TLI, CFI >=.95, SRMR<.08). Education had a positive direct effect on cognitive function (standardized direct effect = 0.08, p=0.002). Although the indirect effect of cognitive complexity was non-significant, hazardous occupational environment mediated education’s link to cognitive function (standardized indirect effect= 0.018, p=0.03). A one-unit increase in the hazardous occupational environment was associated with a 0.06 decline in cognitive function accounting for education, gender, race/ethnicity, depressive symptoms, baseline cognition, and age of the latest cognitive assessment. No statistically significant relationships between occupational exposure and ADRD were identified due to the limited waves of the new ADRD measures. Findings may inform interventions addressing occupational hazards to modify the association between early-life education and late-life cognitive outcomes.

